# Highlight: New Solutions and Open Questions in Computational Evolutionary Biology

**DOI:** 10.1093/gbe/evz237

**Published:** 2019-11-07

**Authors:** Casey McGrath

The dawn of the computer and information age in the last century left virtually no field untouched. In biology, computational advances enabled scientists to generate, store, and analyze large-scale data sets that could scarcely have been imagined decades earlier. These advances ultimately led to the publication of the first bacterial genome sequence in 1995 (Fleischmann et al. 1995), and with it, the birth of the genomics era. The advent of high-throughput sequencing further accelerated the pace of data generation to an unprecedented rate. Now, less than a quarter of a century later, genomic data for almost 220,000 individual organisms and another 25,000 metagenomes are currently available through the National Center for Biotechnology Information (NCBI) website, and *Genome Biology and Evolution* has played a role in publishing numerous articles in the field of computational evolutionary biology.

With this wealth of widely available sequence data, the challenge for evolutionary biologists has become how to analyze genomic data sets to answer new questions and reveal new evolutionary insights. Today’s computational biologists do this by applying theoretical methods, mathematical modeling, and computational simulations in novel ways. They advance the field of evolutionary biology by providing tools and methods that can be used to gain insight into evolutionary processes in a variety of systems and at multiple scales.

*Genome Biology and Evolution’*s virtual issue on computational biology highlights some of these new approaches that have been published in the journal over the last 3 years. Some of these computational methods have practical uses in neighboring fields of biology. For example, in their article, “In Silico Identification of Candidate Genes for Fertility Restoration in Cytoplasmic Male Sterile Perennial Ryegrass (*Lolium perenne* L.),” Sykes et al. (2017) present a pipeline for identifying candidate *restorer of fertility* (*Rf*) genes in any plant species. This is a key element in strategies that attempt to use hybrid breeding to increase crop yield, and this method, according to the authors, “provide[s] plant breeders with a molecular tool for candidate *Rf* gene identification and thus facilitate[s] the implementation of hybrid breeding schemes.”

Other articles in the issue present computational tools designed to improve and advance genomic analysis. In “IMPUTOR: Phylogenetically Aware Software for Imputation of Errors in Next-Generation Sequencing,” Jobin et al. (2018) present software that improves the completeness and accuracy of next-generation sequence data. Their method uses phylogenetic information and the principle of parsimony to correct errors and impute missing bases due to low coverage. This is especially important given recent evidence of errors in sequence databases due to DNA damage/mutagenic processes (Chen et al. 2017).

Duchemin et al. (2017) present a tool for genomic analysis on a more macro level in their paper, “DeCoSTAR: Reconstructing the Ancestral Organization of Genes or Genomes Using Reconciled Phylogenies.” Building on earlier work, this software reconstructs putative ancestral states of genomic “adjacencies,” that is, genomic features that are adjacent in the genome. Importantly, these adjacencies can be studied at virtually any level, so that DeCoSTAR can be used for investigating “ancestral domain structures of a modular protein, as well as chromosome organizations of whole ancestral genomes, or fusion/fission histories or modular genes.”

Additional tools that promise to expand the possibilities of genomic analysis include *MultiTwin* (“*MultiTwin*: A Software Suite to Analyze Evolution at Multiple Levels of Organization Using Multipartite Graphs”) from Corel et al. (2018), which allows for the “integration of several levels of biological organization (genes, genomes, communities, environments) [for] more comprehensive analyses of gene sharing and improved sequence-based classifications.” Furthermore, Wang et al. (2017) describe a method for identifying allele-specific gene expression in natural populations in their paper, “Bayesian Inference of Allele-Specific Gene Expression Indicates Abundant Cis-Regulatory Variation in Natural Flycatcher Populations.”

In addition to the above tools, which promise to improve the accuracy, power, and potential of genomic analyses, the virtual issue highlights studies that provide new insight into evolutionary theory and challenge popular beliefs. In “Identifying Drivers of Parallel Evolution: A Regression Model Approach,” Bailey et al. (2018) use statistical models and data from experimentally evolved *Saccharomyces cerevisiae* populations to undertake the first empirical test of the theory that mutation and selection can impact patterns of parallel evolution equally. Furthermore, in their article, “Strategies for Partitioning Clock Models in Phylogenomic Dating: Application to the Angiosperm Evolutionary Timescale,” Foster and Ho (2017) discuss lingering problems with molecular clock studies and the importance of assigning separate molecular clock models to different subsets of the data, a process they term clock-partitioning. The authors show that “judicious clock-partitioning can improve the precision of molecular dating based on phylogenomic data” and demonstrate this by deriving highly precise age estimates for several key nodes in the angiosperm phylogeny.

Lastly, the virtual issue includes two papers that contribute to ongoing discussions in the field regarding appropriate methods and best practices for genomic evolutionary analysis. In “Further Simulations and Analyses Demonstrate Open Problems of Phylostratigraphy,” Moyers and Zhang (2017) discuss the presence of inherent biases in phylostratigraphic results and propose a method for reanalysis that may help reduce spurious findings. In their Letter, “RelTime Relaxes the Strict Molecular Clock throughout the Phylogeny,” Battistuzzi et al. (2018) undertake a data reanalysis to provide deeper insight into the inner workings of their RelTime method, which estimates divergence times when evolutionary rates vary among lineages.

Together, this selection of manuscripts highlights some of the newest techniques and solutions for genomic data analysis, as well as summarizing ongoing areas of debate in the fields of computational biology, genomics, and evolution. *Genome Biology and Evolution* strives to be a resource and a site for continued discourse on topics at the intersection of these fields, in the hopes of advancing greater collaboration and a better understanding of these issues. 
